# Rearrangements, Expression, and Clinical Significance of MYB and MYBL1 in Adenoid Cystic Carcinoma: A Multi-Institutional Study

**DOI:** 10.3390/cancers14153691

**Published:** 2022-07-28

**Authors:** Marta Persson, Mattias K. Andersson, Yoshitsugu Mitani, Margaret S. Brandwein-Weber, Henry F. Frierson, Christopher Moskaluk, Isabel Fonseca, Renata Ferrarotto, Werner Boecker, Thomas Loening, Adel K. El-Naggar, Göran Stenman

**Affiliations:** 1 Sahlgrenska Center for Cancer Research, Department of Pathology, University of Gothenburg, SE-405 30 Gothenburg, Sweden; marta.ma.persson@vgregion.se (M.P.); mattias.andersson@llcr.med.gu.se (M.K.A.); 2 Department of Pathology, The University of Texas MD Anderson Cancer Center, Houston, TX 77030, USA; yomitani@mdanderson.org (Y.M.); anaggar@mdanderson.org (A.K.E.-N.); 3 Department of Pathology, Icahn School of Medicine at Mount Sinai, New York, NY 10019, USA; margaret.brandwein@mountsinai.org; 4 Department of Pathology, University of Virginia Health System, Charlottesville, VA 22908, USA; hff@virginia.edu (H.F.F.J.); cam5p@virginia.edu (C.M.); 5 Serviço de Anatomia Patológica, Instituto Português de Oncologia de Francisco Gentil-Lisboa and Instituto de Anatomia Patológica, Faculdade de Medicina de Lisboa, 1649-028 Lisbon, Portugal; ifonseca@medicina.ulisboa.pt; 6 Department of Thoracic Head and Neck Medical Oncology, The University of Texas MD Anderson Cancer Center, Houston, TX 77030, USA; rferrarotto@mdanderson.org; 7 Gerhard Domagk Institute of Pathology, University of Muenster, 48149 Muenster, Germany; boecker@me.com; 8 Gerhard-Seifert Reference Centre, Hansepathnet, 22453 Hamburg, Germany; loening@uke.uni-hamburg.de

**Keywords:** *MYB*, *MYBL1*, adenoid cystic carcinoma, prognostic biomarker, FISH, immunohistochemistry, tumor suppressor gene

## Abstract

**Simple Summary:**

Adenoid cystic carcinoma (ACC) is an aggressive glandular cancer with poor prognosis that preferentially occurs in the head and neck. The *MYB* and *MYBL1* oncogenes are main oncogenic drivers, but the true frequency and clinical significance of these alterations are unclear. Here, we have used tissue microarrays to study these genes in a multi-institutional study of close to 400 ACCs, the largest study to date. We found alterations of *MYB*/*MYBL1* in 78% of the cases and overexpression of the MYB/MYBL1 proteins in 93% of the cases. Importantly, we show that patients with loss of one part of the *MYB* gene and its neighboring sequences on chromosome 6 have a significantly shorter overall survival compared to those without loss. Our study provides new knowledge about the frequency and clinical significance of *MYB*/*MYBL1* alterations and identifies genes with tumor suppressive functions on chromosome 6 that contribute to poor prognosis in ACC.

**Abstract:**

Adenoid cystic carcinoma (ACC) is an aggressive head and neck malignancy characterized by a t (6;9) translocation resulting in an *MYB–NFIB* gene fusion or, more rarely, an *MYBL1* fusion. The true frequency and clinical significance of these alterations are still unclear. Here, we have used tissue microarrays and analyzed 391 ACCs and 647 non-ACC salivary neoplasms to study the prevalence, expression, and clinical significance of *MYB/MYBL1* alterations by FISH and immunohistochemistry. Alterations of *MYB* or *MYBL1* were found in 78% of the cases, of which 62% had *MYB* alterations and 16% had *MYBL1* rearrangements. Overexpression of MYB/MYBL1 oncoproteins was detected in 93% of the cases. *MYB* split signal, seen in 39% of the cases, was specific for ACC and not encountered in non-ACC salivary tumors. Loss of the 3′-part of *MYB* was enriched in grade 3 tumors and was a significant independent prognostic biomarker for overall survival in multivariate analyses. We hypothesize that loss of the 3′-part of *MYB* results from an unbalanced t(6;9) leading to an *MYB–NFIB* fusion with concomitant loss of the segment distal to the *MYB* breakpoint in 6q23.3. Our study provides new knowledge about the prevalence and clinical significance of *MYB/MYBL1* alterations and indicates the presence of genes with tumor suppressive functions in 6q23.3-qter that contribute to poor prognosis and short overall survival in ACC.

## 1. Introduction

Adenoid cystic carcinoma (ACC) is a rare malignancy that preferentially occurs in the head and neck region [1,2]. It is one of the most common salivary gland cancers, but may also occur at other sites, such as the breast, skin, lung, female genital tract, and prostate [3]. It is often a slow-growing cancer with a protracted clinical course and a poor long-term prognosis [1,2,4,5,6]. About 40% of the patients develop local recurrencies and up to 60% develop distant metastases within 10 years after diagnosis. The standard treatment for resectable ACCs is radical surgery followed by postoperative radiotherapy [2,6,7]. However, so far there are no effective systemic or targeted therapies available for patients with recurrent and/or metastatic disease. There is thus an unmet need for new therapeutic targets and treatment strategies for patients with this fatal cancer.

We have previously identified a recurrent t(6;9)(q23;p24) translocation in ACC [8] and demonstrated that it results in a fusion of the two transcription factor genes *MYB* in 6q23.3 and *NFIB* in 9p23–22.3 [9,10,11,12]. The t(6;9) was, together with the t(X;1) translocation in renal cancer, the first characteristic translocation identified in malignant human epithelial tumors [13]. The MYB–NFIB fusions consist of the N-terminal part of MYB, including the DNA-binding and transcription activation domains, linked to the C-terminal end of NFIB [9]. In a small subset of cases, *MYB* is replaced by the closely related *MYBL1* gene [14,15]. The structure and functional consequences of the *MYBL1–NFIB* fusions are very similar to those of the *MYB* fusions. In addition to gene fusion, *MYB* and *MYBL1* can also be activated by enhancer hijacking with breakpoints located either upstream or downstream of the genes [16].

Although previous studies have unequivocally established that the t(6;9) translocation and *MYB/MYBL1* fusions are the major genomic hallmarks of ACC [9,11,17,18,19,20], the true frequency and clinical significance of these alterations are still unclear. Previous studies have shown that the frequency of rearrangements/expression of MYB varies from 16 to 100%, depending on the method used for detection of *MYB* alterations [21]. The frequency of the less common *MYBL1* rearrangements is still unknown. In routine clinical work, fluorescence in situ hybridization (FISH) and immunohistochemistry (IHC) are the gold standard methods for analyses of such biomarkers. Here, we have used tissue microarrays (TMAs) to study rearrangements, expression, and the clinical significance, of MYB and MYBL1 in a multi-institutional study of ACC, the largest ACC cohort studied to date.

## 2. Materials and Methods

### 2.1. Tumor Material

Formalin-fixed, paraffin-embedded (FFPE) sections from tissue microarrays (TMAs), including 498 ACCs, 1019 non-ACC salivary neoplasms, and 40 non-salivary carcinomas (Supplementary Table S1), were available for analysis [22,23,24]. Each tumor was represented by at least two core biopsies. We also had access to paraffin blocks from 47 ACCs. TMA paraffin sections and tissue blocks were obtained from the Departments of Pathology at University Medical Center Hamburg-Eppendorf, the University of Texas MD Anderson Cancer Center, the University of Alabama at Birmingham, the University of Virginia Health System/Charlottesville, and Instituto Português de Oncologia Francisco Gentil. Survival data was available for 366 cases. In addition, we had access to the following clinicopathological parameters: sex, age, perineural invasion, and tumor grade (Supplementary Table S2). Tumors were graded as lesions with no solid component (grade 1), <30% solid component (grade 2), and ≥30% solid component (grade 3) [25].

### 2.2. Fluorescence In Situ Hybridization (FISH) Analysis

To detect rearrangements of *MYB* and *MYBL1*, FISH was used to analyze TMAs with locus-specific probes for *MYB* (6q23.3; dual-color MYB split probe; Abnova, Taipei, Taiwan) and *MYBL1* (8q13.1; dual-color MYBL1 break-apart probe; Empire Genomics, Buffalo, NY, USA). The protocols for pretreatment, hybridization, and post-hybridization washes were as recommended by the manufacturers. Cell nuclei were stained blue with DAPI. Fluorescence signals were digitized, processed, and analyzed with the CytoVision image-analysis system (Applied Imaging, San Jose, CA, USA) and Isis FISH imaging system v.5.5 (MetaSystems, Altlussheim, Germany). At least 20 nuclei were scored from each core biopsy.

### 2.3. Immunohistochemistry (IHC)

FFPE sections from ACC TMAs and tumor blocks were deparaffinized, and antigen epitopes were retrieved with EnVision FLEX Target Retrieval Solution pH 9 (Agilent Dako, Santa Clara, CA, USA). The slides were rinsed, and endogenous peroxidase activity was blocked with the EnVision Flex Mini Kit (Agilent Dako) according to the manufacturer’s instructions. Slides were incubated at room temperature with an MYB monoclonal antibody (SPM175; Santa Cruz Biotechnology, Dallas, TX, USA). Bound antibodies were detected with an HRP-conjugated secondary antibody and visualized with EnVision FLEX DAB+ Chromogen substrate (Agilent Dako). Cell nuclei were counterstained with hematoxylin. Control sections were treated identically but without the primary antibody. TMA slides were scanned with a MIRAX SCAN microscope (Carl Zeiss, Göttingen, Germany) and Pannoramic SCAN Control software (3DHISTECH, Budapest, Hungary). Images were viewed with the Pannoramic Viewer v1.15.4 (3DHISTECH). MYB immunostaining was considered positive if >20% of tumor cells showed strong nuclear immunoreactivity.

### 2.4. Statistical Analyses

Kaplan–Meier survival analyses and Chi-square tests were conducted with Prism v.9.3.1 (GraphPad Software, San Diego, CA, USA). Uni- and multivariate analyses using Cox regression were performed with SPSS Statistics v.28 (IBM, Armonk, NY, USA). Confirmation of the proportional hazards assumption and the linearity of Martingale and deviance residuals was performed with the survival package of R.

## 3. Results

### 3.1. Genomic Alterations of MYB and MYBL1 in ACC

**Figure 1:**
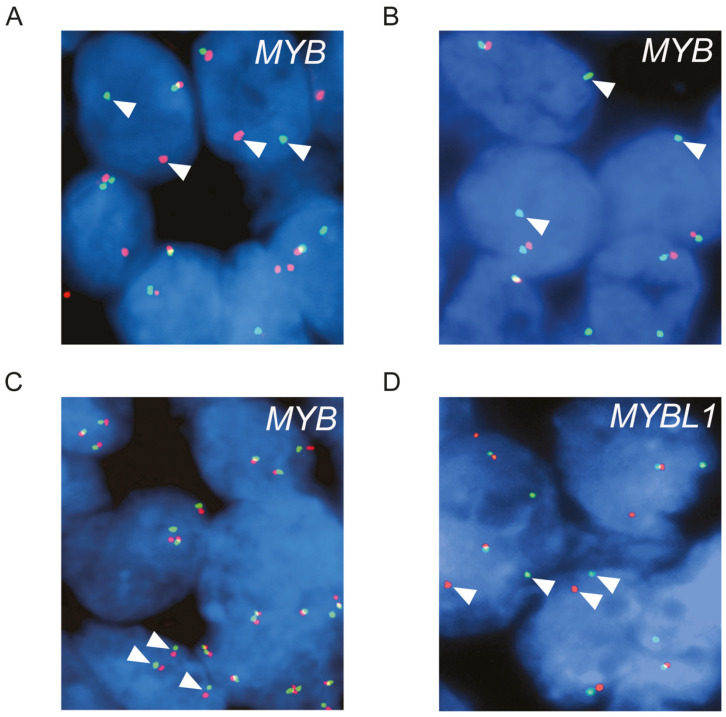
FISH analyses of *MYB* and *MYBL1* in ACC. (**A**) ACC with a split *MYB* signal (separated red and green signals indicated by arrowheads). (**B**) ACC with loss of the 3′-part of *MYB* (loss of one red signal; arrowheads indicate the remaining green signal). (**C**) ACC with gain of one copy of *MYB* (three fused red/green signals indicated by arrowheads). (**D**) ACC with a split *MYBL1* signal (separated red and green signals indicated by arrowheads). Images were captured using an 100× objective.

**Figure 2:**
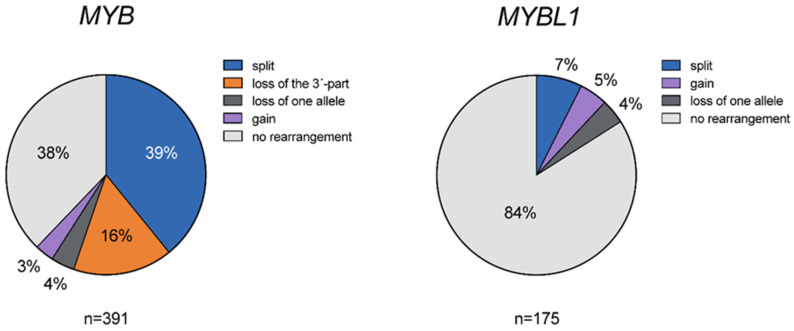
*MYB* and *MYBL1* alterations in ACC. Pie charts showing the frequencies of different types of *MYB* and *MYBL1* alterations detected by FISH in ACC.

To study the frequency and types of alterations of *MYB* in ACC, we analyzed 391 tumors using FISH (Supplementary Table S1). Alterations of the *MYB* locus were detected in 62.1% of the tumors (Figure 1A–C and Figure 2). Split signals, indicating translocation of *MYB*, were found in 39.1% (153/391) (Figure 1A). In contrast, split signals were not detected in any of the 647 analyzable non-ACC salivary tumors or 32 non-salivary carcinomas, indicating that *MYB* split is specific for ACC. Loss of the 3′-part of *MYB* was seen in 16.1% (63/391) (Figure 1B) of the tumors, and gain of an apparently intact *MYB* allele was seen in 3.1% (12/391) (Figure 1C). In addition, loss of one *MYB* allele was seen in 3.8% (15/391). In the remaining 37.9% of the tumors (148/391), no alterations of the *MYB* locus were detected by the FISH split probe used.

Alterations of the *MYBL1* locus were found in 16% (28/175) of the ACCs (Figure 1 D and Figure 2). Split signals indicating translocation of *MYBL1* were detected in 7.4% (13/175), gain of one apparently intact *MYBL1* allele in 4.6% (8 of 175), and loss of one *MYBL1* allele in 4% (7/175) (Figure 1D). All tumors with rearrangements of *MYBL1* were *MYB* negative by FISH.

### 3.2. Expression of MYB and MYBL1 Oncoproteins in ACC

**Figure 3:**
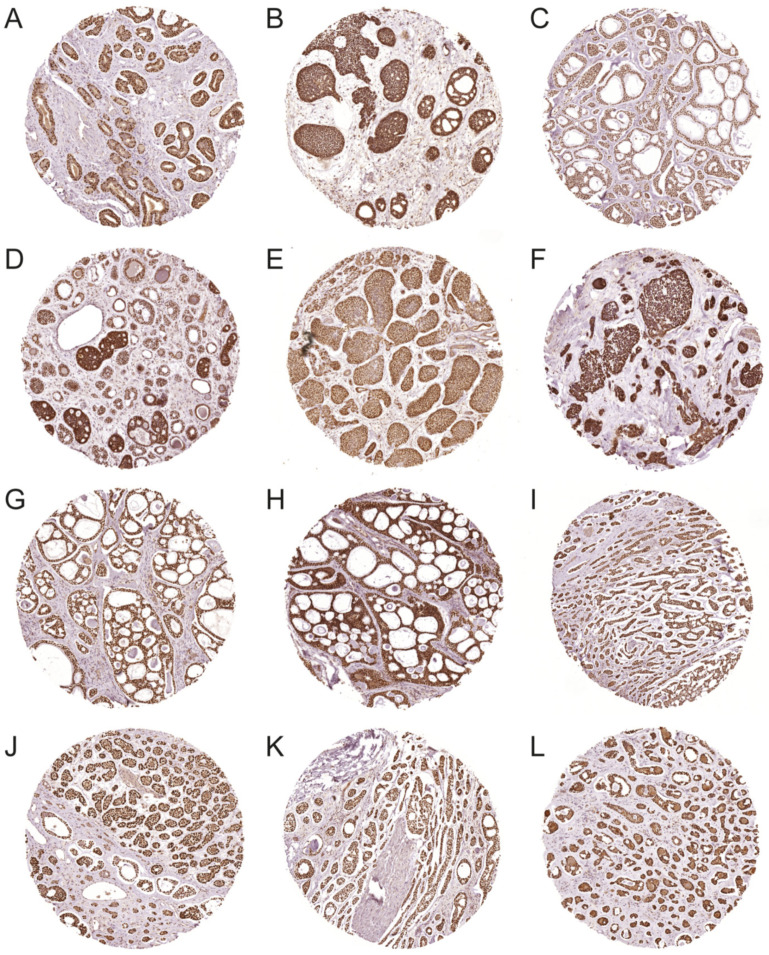
IHC staining of the MYB and MYBL1 oncoproteins in ACC tissue microarrays. (**A**–**C**) MYB staining in three ACCs with *MYB* split signals. (**D**–**F**) MYB staining in three ACCs with loss of the 3′-part of *MYB*. (**G**) MYB staining in an ACC with gain of one copy of *MYB*. (**H**,**I**) MYBL1 staining in two ACCs with *MYBL1* split signals (no *MYB* rearrangements by FISH). (**J**–**L**) MYB/MYBL1 staining in three ACCs with no rearrangements of *MYB* or *MYBL1* by FISH. Images were captured using a 20× objective.

The expression of the MYB oncoprotein was studied by IHC in TMAs and tissue sections containing 292 ACCs (Figure 3). Strong nuclear staining for MYB was found in 93.2% (272/292) of the ACCs (Figure 3A–L). The remaining tumors were all negative. Normal tissues adjacent to the tumors were also consistently negative. All tumors that were *MYBL1*-positive and *MYB*-negative by FISH (*n* = 11) stained positive for the MYB antibody (Figure 3H,I), indicating that the MYB antibody reacts with both MYB and the closely related MYBL1 oncoprotein. MYB and MYBL1 were overexpressed in all morphological subtypes of ACC, including tubular, cribriform, solid, and mixed forms of these patterns (Figure 3A–L). In tumors with tubular differentiation, mainly the outer myoepithelial cells were positive for MYB. Analysis of whole tissue sections from 47 ACCs revealed limited heterogeneity in the staining pattern between different tumors. Taken together, our findings clearly show that more than 90% of ACCs are MYB/MYBL1-positive, demonstrating that MYB/MYBL1 is a significant biomarker for ACC.

### 3.3. Clinical Significance of MYB and MYBL1 Rearrangements

**Figure 4:**
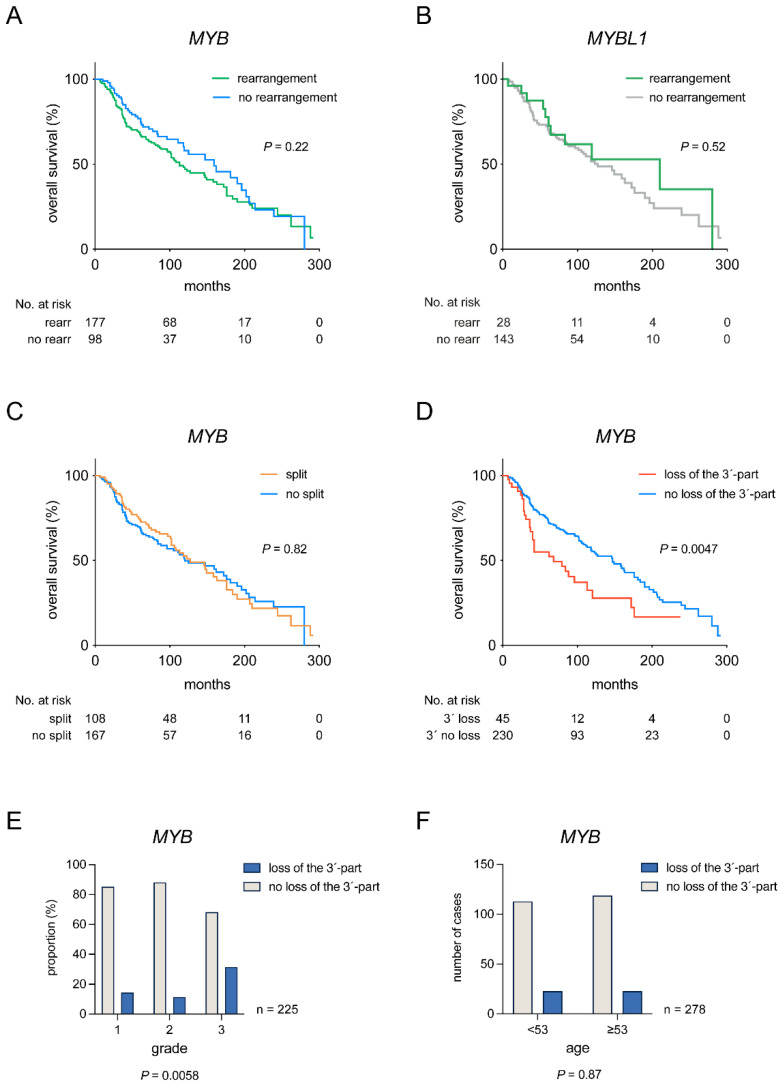
Prognostic significance of *MYB* and *MYBL1* alterations in ACC. (**A**) OS in ACC patients with or without *MYB* rearrangements. (**B**) OS in ACC patients with or without *MYBL1* rearrangements. (**C**) OS of ACC patients with or without *MYB* split signal. (**D**) OS of ACC patients with or without loss of the 3′-part of *MYB*. (**E**) Distribution of ACC cases with or without loss of the 3′-part of *MYB* by tumor grade. (**F**) Distribution of ACC cases with or without loss of the 3′-part of *MYB* by median age.

To study the clinical significance of *MYB*/*MYBL1* rearrangements, we analyzed the overall survival (OS) of ACC patients with and without these rearrangements (Figure 4A,B). OS did not differ in patients with or without rearrangement of *MYB* (*p* = 0.22) and *MYBL1* (*p* = 0.52) (Figure 4A,B). To further investigate a potential clinical impact of specific *MYB* rearrangements, we sequentially compared patients harboring one of the two most common *MYB* rearrangements, *MYB* split and loss of the 3′-part of *MYB*, with patients lacking such rearrangements (Table 1 and Figure 4C,D). Whereas *MYB* split did not provide prognostic information, loss of the 3′-part of *MYB* was significantly associated with shorter OS (5-year OS, 55% vs. 74%; 10-year OS, 28% vs. 55%). Further analyses revealed that loss of the 3′-part of *MYB* was present in tumors of all grades but was significantly more common in grade 3 tumors (Figure 4E). Loss of the 3′-part was not associated with patient age at diagnosis (Figure 4F), sex, or perineural invasion (data not shown). Neither was *MYB* split associated with any of the analyzed clinicopathological parameters (data not shown). Notably, loss of the 3′-part of *MYB* was a significant independent prognostic biomarker for OS in multivariate analyses together with age and grade (Table 1).

## 4. Discussion

Previous studies have unequivocally demonstrated that constitutive activation of *MYB*, or more rarely *MYBL1*, are key genomic events in the pathogenesis of ACC [9,11,14,15,17,18,19,20]. However, the actual frequency of *MYB/MYBL1* alterations and overexpression is still unclear. From a diagnostic point of view, this is particularly important to clarify since these genes are increasingly used as ancillary markers for ACC. In a recent systematic review and meta-analysis of the prevalence and prognostic impact of the t(6;9) and *MYB–NFIB* fusion in head and neck ACC, the prevalence varied significantly from 16–100% depending on the methodology used in the different studies [21]. In the largest studies, *MYB* rearrangements or *MYB–NFIB* fusions were detected in 33–75% of the ACCs by FISH (*n* = 24–100) [18,26,27,28,29,30,31,32]. The corresponding figures for *MYBL1* rearrangements/fusions using FISH varied from 9 to 23% (*n* = 33–100) [30,31,32]. 

The present FISH analysis revealed rearrangements/gain of *MYB* in 62% (243/391) of the ACCs and of *MYBL1* in 16% (28/175). Thus, our FISH assays detected alterations of *MYB* or *MYBL1* in 78% of the ACCs. Notably, IHC revealed overexpression of MYB or MYBL1 oncoproteins in 93.2% of the 292 ACCs analyzed. This frequency is almost identical to a recent RNA in situ hybridization (ISH) study showing *MYB* overexpression in 92% of 77 analyzed ACCs [33]. In addition, the *MYB* ISH study showed an 89% specificity for ACC compared to a 54% specificity for MYB IHC. Although not as specific as ISH, IHC is a method available in pathology laboratories worldwide. The discrepancy between the frequency of *MYB/MYBL1* alterations by FISH and MYB/MYBL1 oncoprotein expression likely reflects different mechanisms of activation of *MYB/MYBL1*, including gene fusion, gain/amplification, and enhancer hijacking. All in all, our study, the largest to date, clearly demonstrates that activation of *MYB*/*MYBL1* is nearly universal in ACC. Future studies will reveal whether there are other unknown drivers in *MYB/MYBL1* negative ACCs or whether these instead may be ACC mimics. 

The most common *MYB* alteration in our study was *MYB* split in 39.1% of the cases, followed by loss of the 3´-part of *MYB* in 16.1%, loss of one *MYB* allele in 3.8%, and gain of one *MYB* allele in 3.1%. The corresponding figures for *MYBL1* alterations were *MYBL1* split in 7.4%, gain of *MYBL1* in 4.6%, and loss of one *MYBL1* allele in 4%. Importantly, *MYB* split signals were not seen in any of the more than 600 non-ACC salivary tumors, demonstrating that *MYB* FISH split signals are indeed specific for ACC. Based on previous cytogenetic and arrayCGH studies [17,34], gain of one *MYB* allele is likely to result from gene duplication rather than trisomy 6 since this aberration has previously not been detected in ACC [34]. Gene duplication has previously been shown as a mechanism of *MYB* activation in T-cell acute lymphoblastic leukemia [35]. In contrast, gain of one *MYBL1* allele is more likely to result from trisomy 8, which is a known recurrent aberration in ACC [34]. Importantly, there was no difference in MYB protein expression between ACCs with different genomic alterations, thus confirming that all these changes result in activation of MYB/MYBL1 expression.

There is partly contradicting information in the literature whether the *MYB* fusion carries prognostic information. Although some studies have suggested certain correlations between *MYB* alterations and various clinicopathological parameters, most have failed to find significant associations [21,36]. For instance, Rettig et al. [28] have suggested that minor salivary gland ACCs are more often *MYB* fusion-positive and that fusions are more common in females than in males. Mitani et al. [15] found that ACCs with *MYB* alterations are associated with recurrences and metastases. High *MYB* [27] or *MYB/MYBL1* [14] mRNA expression in ACC has also been linked to poor patient survival, solid tumor histology, and advanced disease stage. In the present study, we found no significant differences in OS between ACC patients with or without rearrangements of *MYB* or *MYBL1*. However, detailed sequential comparison of patients with and without the two most common types of *MYB* rearrangements revealed that loss of the 3′-part of *MYB* was significantly associated with shorter OS. Importantly, loss of the 3´-part of *MYB* was also an independent prognostic marker for OS in multivariate analyses and was significantly enriched in grade 3 tumors. In contrast, *MYB* split did not correlate with OS or any other clinicopathological parameter.

Our finding of loss of the 3′-part of *MYB* and flanking sequences is strongly supported by previous cytogenetic and molecular observations of deletions of the terminal part of 6q in a subset of ACCs [8,17,37,38,39,40,41]. The present and previous observations indicate that there are one or more genes in 6q23.3-qter with tumor suppressive functions and whose loss/losses contribute to poor prognosis and short OS in ACC. We hypothesize that the loss of the 3′-part of *MYB* results from an unbalanced 6;9-translocation leading to an *MYB–NFIB* fusion with concomitant loss of the 6q-segment distal to the *MYB* breakpoint in 6q23.3. Further molecular analyses will reveal whether any of the known tumor suppressors in this region, e.g., *LATS1*, *PARK2*, or *PLAGL1* [42,43,44,45,46], is the target of these deletions in ACC. 

A limitation of our study is that we have only used *MYB/MYBL1* break-apart FISH probes and MYB IHC, and we have not screened for fusions by RT-PCR or fusion-specific FISH. Moreover, our FISH assays do not detect breakpoints not covered by our *MYB* and *MYBL1* probes, such as cases in which the genes are activated by enhancer hijacking. However, these cases may be readily identified by MYB IHC. Although not specifically addressed in the present study, MYB overexpression does not seem to be limited to ACC but may occasionally occur also in other salivary gland tumors, such as acinic cell carcinoma [47], and basal cell and myoepithelial neoplasms [3,33].

## 5. Conclusions

This comprehensive multi-institutional study, the largest to date, demonstrates that alterations in *MYB* and *MYBL1* occur in 78% of ACCs and that overexpression of the MYB/MYBL1 oncoproteins is found in 93% of the cases. These findings further strengthen *MYB*/*MYBL1* as significant diagnostic biomarkers and targets for therapy in ACC. Moreover, we show that an *MYB* split signal is specific for ACC and that loss of the 3′-part of *MYB* is enriched in grade 3 tumors. Notably, we also demonstrate that loss of the 3′-part of *MYB* and its flanking sequences is an independent prognostic marker for OS in multivariate analyses. Taken together, our study provides new knowledge about the prevalence and clinical significance of *MYB* and *MYBL1* alterations in ACC and indicates the presence of genes with tumor suppressive functions in 6q23.3-qter that contribute to poor prognosis in a subset of tumors.

## Figures and Tables

**Table 1:** Univariate and multivariate (*n* = 224) analyses of the hazard function of death in ACC patients.

		Univariate			Multivariate	
	HR	HR 95% CI	*p*-Value	HR	HR 95% CI	*p*-Value
*MYB*—loss of the 3′-part (*n* = 278)	1.791	1.187–2.703	0.006	1.633	1.032–2.584	0.036
*MYB*—split (*n* = 278)	0.962	0.686–1.349	0.822			
Age (*n* = 365)	1.027	1.017–1.038	<0.001	1.028	1.014–1.043	<0.001
Sex (*n* = 366)	1.110	0.827–1.491	0.487			
Perineural invasion (*n* = 180)	0.770	0.397–1.492	0.439			
Grade (*n* = 304)						
1 (*n* = 127)	reference			reference		
2 (*n* = 104)	1.369	0.904–2.071	0.138	1.440	0.886–2.339	0.141
3 (*n* = 73)	3.751	2.480–5.673	<0.001	4.481	2.760–7.275	<0.001

HR, hazard ratio; CI, confidence interval.

## Data Availability

Data is maintained in this article. Data is not publicly available due to privacy.

## Supplementary Materials

The following supporting information can be downloaded at: https://www.mdpi.com/article/10.3390/cancers14153691/s1, Table S1: Salivary and non-salivary gland tumors included in the tissue microarrays (TMAs) and analyzed by fluorescence in situ hybridization (FISH); Table S2: Clinicopathological characteristics of 366 adenoid cystic carcinomas.

## References

[1] Stenman G., Licitra L., Said-Al-Naief N., van Zante A., Yarbrough W.G., El-Naggar A.K., Chan J.K.C., Grandis J.R., Takata T., Slootweg P.J. (2017). Adenoid cystic carcinoma. World Health Organization Classification of Head and Neck Tumours.

[2] Coca-Pelaz A., Rodrigo J.P., Bradley P.J., Poorten V.V., Triantafyllou A., Hunt J.L., Strojan P., Rinaldo A., Haigentz M., Takes R.P. (2015). Adenoid cystic carcinoma of the head and neck—An update. Oral Oncol..

[3] Brill L.B., Kanner W.A., Fehr A., Andren Y., Moskaluk C.A., Loning T., Stenman G., Frierson H.F. (2011). Analysis of MYB expression and MYB-NFIB gene fusions in adenoid cystic carcinoma and other salivary neoplasms. Mod. Pathol..

[4] Laurie S.A., Ho A.L., Fury M.G., Sherman E., Pfister D.G. (2011). Systemic therapy in the management of metastatic or locally recurrent adenoid cystic carcinoma of the salivary glands: A systematic review. Lancet Oncol..

[5] Xu B., Drill E., Ho A., Ho A., Dunn L., Prieto-Granada C.N., Chan T., Ganly I., Ghossein R., Katabi N. (2017). Predictors of Outcome in Adenoid Cystic Carcinoma of Salivary Glands: A Clinicopathologic Study with Correlation Between MYB Fusion and Protein Expression. Am. J. Surg. Pathol..

[6] Carlson J., Licitra L., Locati L., Raben D., Persson F., Stenman G. (2013). Salivary Gland Cancer: An Update on Present and Emerging Therapies. Am. Soc. Clin. Oncol. Educ. Book.

[7] Geiger J.L., Ismaila N., Beadle B., Caudell J.J., Chau N., Deschler D., Glastonbury C., Kaufman M., Lamarre E., Lau H.Y. (2021). Management of Salivary Gland Malignancy: ASCO Guideline. J. Clin. Oncol..

[8] Stenman G., Sandros J., Dahlenfors R., Juberg-Ode M., Mark J. (1986). 6q- and loss of the Y chromosome—Two common deviations in malignant human salivary gland tumors. Cancer Genet. Cytogenet..

[9] Persson M., Andren Y., Mark J., Horlings H.M., Persson F., Stenman G. (2009). Recurrent fusion of MYB and NFIB transcription factor genes in carcinomas of the breast and head and neck. Proc. Natl. Acad. Sci. USA.

[10] Andersson M.K., Stenman G. (2016). The landscape of gene fusions and somatic mutations in salivary gland neoplasms—Implications for diagnosis and therapy. Oral Oncol..

[11] Andersson M.K., Afshari M.K., Andrén Y., Wick M.J., Stenman G. (2017). Targeting the Oncogenic Transcriptional Regulator MYB in Adenoid Cystic Carcinoma by Inhibition of IGF1R/AKT Signaling. J. Natl. Cancer Inst..

[12] Andersson M.K., Åman P., Stenman G. (2019). IGF2/IGF1R Signaling as a Therapeutic Target in MYB-Positive Adenoid Cystic Carcinomas and Other Fusion Gene-Driven Tumors. Cells.

[13] Mertens F., Johansson B., Fioretos T., Mitelman F. (2015). The emerging complexity of gene fusions in cancer. Nat. Cancer.

[14] Brayer K.J., Frerich C.A., Kang H., Ness S.A. (2016). Recurrent Fusions in *MYB* and *MYBL1* Define a Common, Transcription Factor–Driven Oncogenic Pathway in Salivary Gland Adenoid Cystic Carcinoma. Cancer Discov..

[15] Mitani Y., Liu B., Rao P.H., Borra V.J., Zafereo M., Weber R.S., Kies M., Lozano G., Futreal P.A., Caulin C. (2016). Novel *MYBL1* Gene Rearrangements with Recurrent *MYBL1–NFIB* Fusions in Salivary Adenoid Cystic Carcinomas Lacking t(6;9) Translocations. Clin. Cancer Res..

[16] Drier Y., Cotton M.J., Williamson K.E., Gillespie S.M., Ryan R.J.H., Kluk M.J., Carey C.D., Rodig S.J., Sholl L.M., Afrogheh A.H. (2016). An oncogenic MYB feedback loop drives alternate cell fates in adenoid cystic carcinoma. Nat. Genet..

[17] Persson M., Andrén Y., Moskaluk C.A., Frierson H.F., Cooke S.L., Futreal P.A., Kling T., Nelander S., Nordkvist A., Persson F. (2012). Clinically significant copy number alterations and complex rearrangements of *MYB* and *NFIB* in head and neck adenoid cystic carcinoma. Genes Chromosom. Cancer.

[18] Ho A.S., Kannan K., Roy D.M., Morris L.G.T., Ganly I., Katabi N., Ramaswami D., Walsh L., Eng S., Huse J.T. (2013). The mutational landscape of adenoid cystic carcinoma. Nat. Genet..

[19] Stephens P.J., Davies H.R., Mitani Y., Van Loo P., Shlien A., Tarpey P.S., Papaemmanuil E., Cheverton A., Bignell G.R., Butler A.P. (2013). Whole exome sequencing of adenoid cystic carcinoma. J. Clin. Investig..

[20] Rettig E.M., Talbot C.C., Sausen M., Jones S., Bishop J.A., Wood L.D., Tokheim C., Niknafs N., Karchin R., Fertig E.J. (2016). Whole-Genome Sequencing of Salivary Gland Adenoid Cystic Carcinoma. Cancer Prev. Res..

[21] De Almeida-Pinto Y.D., Costa S.F.D.S., de Andrade B.A.B., Altemani A., Vargas P.A., Abreu L.G., Fonseca F.P. (2019). t(6;9)(MYB-NFIB) in head and neck adenoid cystic carcinoma: A systematic review with meta-analysis. Oral Dis..

[22] Clauditz T.S., Gontarewicz A., Lebok P., Tsourlakis M.-C., Grob T.J., Muenscher A., Sauter G., Bokemeyer C., Knecht R., Wilczak W. (2012). Epidermal growth factor receptor (EGFR) in salivary gland carcinomas: Potentials as therapeutic target. Oral Oncol..

[23] Clauditz T.S., Gontarewicz A., Wang C.-J., Münscher A., Laban S., Tsourlakis M.C., Knecht R., Sauter G., Wilczak W. (2012). 11q21 Rearrangement is a Frequent and Highly Specific Genetic Alteration in Mucoepidermoid Carcinoma. Diagn. Mol. Pathol..

[24] Clauditz T.S., Gontarewicz A., Bokemeyer C., Sauter G., Knecht R., Münscher A., Wilczak W. (2013). Abundant expression of mTOR kinase in salivary gland tumors—Potentials as therapy target?. J. Oral Pathol. Med..

[25] Szanto P.A., Luna M.A., Tortoledo M.E., White R.A. (1984). Histologic grading of adenoid cystic carcinoma of the salivary glands. Cancer.

[26] Mitani Y., Li J., Rao P.H., Zhao Y.-J., Bell D., Lippman S.M., Weber R.S., Caulin C., El-Naggar A.K. (2010). Comprehensive Analysis of the *MYB-NFIB* Gene Fusion in Salivary Adenoid Cystic Carcinoma: Incidence, Variability, and Clinicopathologic Significance. Clin. Cancer Res..

[27] Mitani Y., Rao P.H., Futreal P.A., Roberts D.B., Stephens P.J., Zhao Y.-J., Zhang L., Mitani M., Weber R.S., Lippman S.M. (2011). Novel Chromosomal Rearrangements and Break Points at the t(6;9) in Salivary Adenoid Cystic Carcinoma: Association with *MYB–NFIB* Chimeric Fusion, *MYB* Expression, and Clinical Outcome. Clin. Cancer Res..

[28] Rettig E.M., Tan M., Ling S., Yonescu R., Bishop J.A., Fakhry C., Ha P.K. (2015). MYB rearrangement and clinicopathologic characteristics in head and neck adenoid cystic carcinoma. Laryngoscope.

[29] Ho A.L., Dunn L., Sherman E.J., Fury M.G., Baxi S.S., Chandramohan R., Dogan S., Morris L.G.T., Cullen G., Haque S. (2016). A phase II study of axitinib (AG-013736) in patients with incurable adenoid cystic carcinoma. Ann. Oncol..

[30] Fujii K., Murase T., Beppu S., Saida K., Takino H., Masaki A., Ijichi K., Kusafuka K., Iida Y., Onitsuka T. (2017). *MYB*, *MYBL1*, *MYBL2* and *NFIB* gene alterations and MYC overexpression in salivary gland adenoid cystic carcinoma. Histopathology.

[31] Andreasen S., Tan Q., Agander T.K., Steiner P., Bjørndal K., Høgdall E., Larsen S.R., Erentaite D., Olsen C.H., Ulhøi B.P. (2018). Adenoid cystic carcinomas of the salivary gland, lacrimal gland, and breast are morphologically and genetically similar but have distinct microRNA expression profiles. Mod. Pathol..

[32] Togashi Y., Dobashi A., Sakata S., Sato Y., Baba S., Seto A., Mitani H., Kawabata K., Takeuchi K. (2018). *MYB* and *MYBL1* in adenoid cystic carcinoma: Diversity in the mode of genomic rearrangement and transcripts. Mod. Pathol..

[33] Rooper L.M., Lombardo K.A., Oliai B.R., Ha P.K., Bishop J.A. (2020). MYB RNA In Situ Hybridization Facilitates Sensitive and Specific Diagnosis of Adenoid Cystic Carcinoma Regardless of Translocation Status. Am. J. Surg. Pathol..

[34] Mitelman F., Johansson B., Mertens F. (2022). Mitelman Database of Chromosome Aberrations and Gene Fusions in Cancer. https://mitelmandatabase.isb-cgc.org.

[35] Lahortiga I., De Keersmaecker K., Van Vlierberghe P., Graux C., Cauwelier B., Lambert F., Mentens N., Beverloo H.B., Pieters R., Speleman F. (2007). Duplication of the MYB oncogene in T cell acute lymphoblastic leukemia. Nat. Genet..

[36] Liu X., Chen D., Lao X., Liang Y. (2019). The value of MYB as a prognostic marker for adenoid cystic carcinoma: Meta-analysis. Head Neck.

[37] Sandros J., Mark J., Happonen R.P., Stenman G. (1988). Specificity of 6q- markers and other recurrent deviations in human malignant salivary gland tumors. Anticancer Res..

[38] Sandros J., Stenman G., Mark J. (1990). Cytogenetic and molecular observations in human and experimental salivary gland tumors. Cancer Genet. Cytogenet..

[39] Queimado L., Reis A., Fonseca I., Martins C., Lovett M., Soares J., Parreira L. (1998). A refined localization of two deleted regions in chromosome 6q associated with salivary gland carcinomas. Oncogene.

[40] Rutherford S., Yu Y., Rumpel C.A., Frierson H.F., Moskaluk C.A. (2006). Chromosome 6 deletion and candidate tumor suppressor genes in adenoid cystic carcinoma. Cancer Lett..

[41] von Holstein S.L., Fehr A., Persson M., Therkildsen M.H., Prause J.U., Heegaard S., Stenman G. (2013). Adenoid Cystic Carcinoma of the Lacrimal Gland: MYB Gene Activation, Genomic Imbalances, and Clinical Characteristics. Ophthalmology.

[42] Abdollahi A., Roberts D., Godwin A.K., Schultz D.C., Sonoda G., Testa J.R., Hamilton T.C. (1997). Identification of a zinc-finger gene at 6q25: A chromosomal region implicated in development of many solid tumors. Oncogene.

[43] Abdollahi A. (2006). LOT1 (ZAC1/PLAGL1) and its family members: Mechanisms and functions. J. Cell. Physiol..

[44] Veeriah S., Taylor B.S., Meng S., Fang F., Yilmaz E., Vivanco I., Janakiraman M., Schultz N., Hanrahan A.J., Pao W. (2009). Somatic mutations of the Parkinson’s disease–associated gene PARK2 in glioblastoma and other human malignancies. Nat. Genet..

[45] Visser S., Yang X. (2010). LATS tumor suppressor: A new governor of cellular homeostasis. Cell Cycle.

[46] Sharif A.A.D., Hergovich A. (2018). The NDR/LATS protein kinases in immunology and cancer biology. Semin. Cancer Biol..

[47] Lee D.Y., Brayer K.J., Mitani Y., Burns E.A., Rao P.H., Bell D., Williams M.D., Ferrarotto R., Pytynia K.B., El-Naggar A.K. (2020). Oncogenic Orphan Nuclear Receptor NR4A3 Interacts and Cooperates with MYB in Acinic Cell Carcinoma. Cancers.

