# Supplementary material for: Rearrangements, Expression, and Clinical Significance of MYB and MYBL1 in Adenoid Cystic Carcinoma: A Multi-Institutional Study
Source: Cancers (Basel). 2022 Jul 28;14(15):3691. doi: 10.3390/cancers14153691 (PMC9367430; doi:10.3390/cancers14153691)
Supplement: Supplementary file 1 [file cancers-14-03691-s001.zip › cancers-1796000-supplementary.pdf]

Supplementary Materials: Rearrangements, Expression, and Clinical Significance of MYB and MYBL1 in Adenoid Cystic Carcinoma: A Multi-Institutional Study

Marta Persson, Mattias K. Andersson, Yoshitsugu Mitani, Margaret S. Brandwein-Weber, Henry F. Frierson Jr., Christopher Moskaluk, Isabel Fonseca, Renata Ferrarotto, Werner Boecker, Thomas Loening, Adel K. El-Naggar and Göran Stenman

Table S1. Salivary and non-salivary gland tumors included in the tissue microarrays (TMAs) and analyzed by fluorescence in situ hybridization (FISH).

Tumor type	No. of cases in the TMAs	No. of cases analyzed by FISH
**Tissue microarrays**		
**Salivary gland neoplasms**		
**Adenoid cystic carcinoma**	498	391
**Mucoepidermoid carcinoma**	343	195
**Acinic cell carcinoma**	181	119
**Adenocarcinoma NOS**	109	63
**Epithelial-myoepithelial carcinoma**	53	41
**Polymorphous adenocarcinoma**	41	32
**Basal cell adenocarcinoma**	25	18
**Cystic adenocarcinoma**	14	11
**Myoepithelial carcinoma**	21	17
**Oncocytic carcinoma**	12	6
**Salivary duct carcinoma**	15	10
**Pleomorphic adenoma**	53	34
**Myoepithelioma**	11	8
**Basal cell adenoma**	86	53
**Warthin tumor**	55	40
**Non-salivary carcinomas**		
**Prostate carcinoma**	10	8
**Mammary carcinoma**	10	5
**Lung adenocarcinoma**	10	9
**Colon carcinoma**	10	10
**Total number of tumors**	**1557**	**1070**

Table S2. Clinicopathological characteristics of 366 adenoid cystic carcinomas.

Parameter	No. of Cases
**Sex**	366
**female**	204 (56%)
**male**	162 (44%)
**Age**	365 (mean 54, range 15-92)
**Perineural invasion**	180
**yes**	158 (88%)
**no**	22 (12%)
**Tumor grade**	304
**1**	127 (42%)
**2**	104 (34%)
**3**	73 (24%)
